# Lys39-Lysophosphatidate Carbonyl Oxygen Interaction Locks LPA_1_ N-terminal Cap to the Orthosteric Site and partners Arg124 During Receptor Activation

**DOI:** 10.1038/srep13343

**Published:** 2015-08-13

**Authors:** Olaposi I. Omotuyi, Jun Nagai, Hiroshi Ueda

**Affiliations:** 1 From the Department of Pharmacology and Therapeutic Innovation, Graduate School of Biomedical Sciences, Nagasaki University, Japan; 2 From the Center for Drug Discovery and Therapeutic Innovation, Nagasaki University, Japan.

## Abstract

Lysophosphatidic acid (LPA) receptor 1 (LPA_1_) is a member of the G protein-coupled receptors mediating the biological response to LPA species. Lack of detailed mechanism underlying LPA/LPA_1_ interaction has hampered the development of specific antagonists. Here, novel N-terminal Lys39 has been identified as a key residue during LPA-type agonist binding and LPA_1_ activation. Analysis of the molecular dynamics (MD) trajectories showed that LPA-type agonist but not VPC-32183 (antagonist) evolved structures with classical GPCR activation signatures such as reduced cytoplasmic transmembrane (TM) 3/TM6 dynamic network, ruptured ionic lock, and formation of a continuous and highly ordered internal water pathway was also observed. In activated state, LPA-type agonists interact with Arg124 (R3.28), Gln125 (Q3.29), Lys294 (K7.36) and a novel N-terminal Lys39. Site-directed mutagenesis showed complete loss of intracellular calcium mobilization in B103 cells expressing R3.28A and Lys39Ala when treated with LPA-type agonists. Structurally, LPA-type agonist via Carbonyl-oxygen/Lys39 interaction facilitated the formation of a hypothetical N-terminal cap tightly packed over LPA_1_ heptahelical bundle. This packing may represent a key mechanism to distinguish an apo-receptor from bound LPA_1_.

Agonist binding to G protein coupled receptors (GPCRs) initiates the transduction of agonist-encoded signals across the biological membrane via the topological rearrangement of GPCRs’ heptahelical domain, recruitment and activation of the intracellular membrane-associated heterotrimeric G (Gα-GDP/βγ) protein complex in a GDP-GTP exchange dependent manner^1^. Gα-GTP subunit ultimately commits to the intracellular cascade following the activation of downstream effector proteins such as phospholipase C (PLC)^2^ and adenylate cyclase (AC)^3^.

Lysophosphatidic acid receptor 1 (LPA_1_) is the second member of the endothelial differentiation gene (EDG) GPCR family^4^ and one of the six LPA receptors (others include LPA_2~6_) mediating the diverse biological activities of lysophosphatidatic acid (LPA) species^4^,^5^. Interest in LPA/LPA_1_ has soared in recent times due to increasing research reports that linked dysfunctional LPA/LPA_1_ signaling to pathological conditions such as fetal hydrocephalus, cardiovascular diseases^6^,^7^,^8^, prostate cancer^9^, dermal fibrosis^10^, rheumatoid arthritis^11^,^12^,^13^ and neuropathic pain^14^,^15^. Interestingly, most of the pathologies are directly associated with increased LPA production or aggressive LPA_1_ expression^9^,^11^,^12^,^13^. LPA_1_ antagonists and chemical inhibitors of enzymes along LPA synthesis pathway have shown promise in the management of the conditions^10^,^16^,^17^ whilst some of the chemical agents are at various developmental or clinical testing stages (e.g. AM095)^16^,^17^.

Arguably, the most daunting challenge to the development of an LPA_1_-specific antagonist is the receptor promiscuity as most EDG receptors share highly conserved orthosteric site residues^18^ and tend to respond to chemicals with similar pharmacophores as observed Ki-16425 and di-octyl glycerol pyrophosphate (DGPP 8:0) with antagonistic activities on LPA_1_~_3_^19^. To solve this problem, drug-development scientists must pay increased attention to contribution of N-terminal residues to ligand recognition and receptor activation; since like most GPCRs, EDG receptors also exhibit low sequence homology/conservation within this region thus, may explain subtle differences in ligand binding and activation of EDG receptors^20^.

In this study, we demonstrate that N-terminal Lys39 is a key partner with previously identified Arg124 (R3.28), Gln125 (Q3.29), and K294 (K7.36) during lysophosphatidic acid (LPA)-type LPA_1_ activation using molecular dynamics (MD) simulation and mutagenesis experiments^21^.

## Results

**Figure 1:**
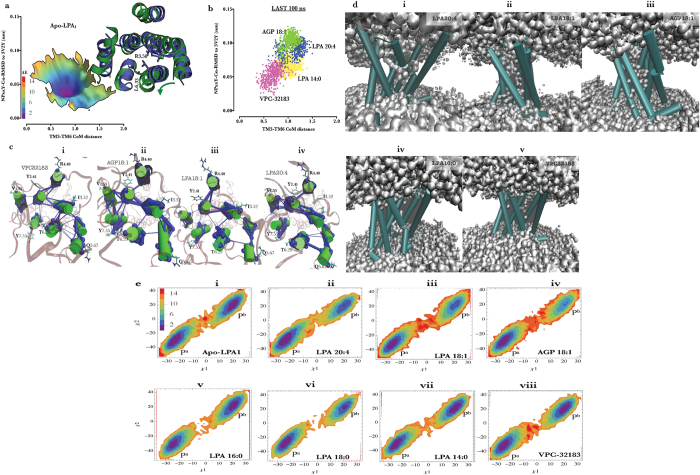
Classical GPCR activation signature in LPA-type agonist bound LPA_1_. (**a**) Free-energy surface of apo-LPA_1_. Inset depicts intermediately activated LPA_1_ (green cartoon) superimposed on the starting LPA_1_ structure (purple cartoon). R3.50 and L6.33 are shown as stick representation. (**b**) Distribution of Cα-NPxxY (N7.49-Y7.53) rmsd from inactive and TM3(Cα-R3.50)-TM6 (Cα-L6.33) distance in structures generated within the last 100. (**c**, *i–iv*) Dynamic network between cytoplasmic ends of transmembrane helices in VPC32183, AGP18:1, LPA18:1 and LPA20:4 bound LPA_1_. (**d**, *i–v)* Average internal water density flow along LPA_1_ bound to strong agonists (LPA20:4, LPA18:1 and AGP18:1), weak agonists (LPA 16:0) and an antagonist (VPC32183). (**e**, *i–viii*) Free-energy surface representation of LPA_1_ P7.50 dihedral angle bound to strong and weak agonists and antagonist. Each dot in “b” represents the mean value of three independent simulations, the green arrow depecits the movement of TM6 away from the ionic lock zone.

The initial LPA_1_ model shared similar seven transmembrane helices conformation with sphingosine 1 phosphate receptor 1 (S1PR1) in complex with an antagonist (PDB ID: 3V2Y)^22^. Since LPA species are LPA_1_ agonist, the initial model was simulated in an apo-state (150 ns) to generate intermediate or active-state features, such as breaking transmembrane (TM) 3-TM6 ionic lock (TM3~TM6 (intracellular) center of mass distance > 1.2 nm) and root mean square deviation (rmsd) of TM7 NPxxY motif from the inactive state (N(7.49)PxxY(7.53) rmsd to 3V2Y > 0.05 nm) as previously observed during β_2_-adrenergic receptor activation^23^. Apo-structures were preferentially trapped in the intermediate state (TM3-TM6 distance ≈ 1.0–1.5 nm, Cα−NPxxY rmsd to 3V2Y ≈ 0.05 nm) (Fig. 1a). Three substructures were harvested from the energy basin (ΔG ≈ 0 Kj/mol, colour bar represents energy) to investigate LPA-dependent LPA_1_ activation. Upon superimposition of one of the three starting structures (green cartoon) on the starting coordinate (purple cartoon), movement of TM6 (R3.50) away (green arrow) from the TM3 (L6.33) relative to the starting model was observed (inset).

### Classical GPCR activation pattern in lysophosphatidic acid-type agonist bound LPA1

Class A GPCR activation is generally characterized by four key events: *rotameric changes in aromatic residue lining the orthosteric site (toggle switch)*^24^, *breaking of TM3-TM6 ionic lock*^23^*, formation of a continuous internal water pathway*^25^*, and kinking of conserved P7.50 (TM7, NPxxY motif)*^26^. Here, we provided evidence LPA-type agonists but not antagonist (VPC-32183 (S)) preferentially drove intermediately activated apo-LPA_1_ to evoke full activation structural signatures in the course of 200 ns simulation time. In LPA_1_/LPA (14:0) complex, LPA_1_ structures generated within the last 100 ns simulation showed fully broken TM3-TM6 ionic lock (CoM distance > 1.2 nm) but NPxxY motif was largely the inactive state (Cα-rmsd from inactive ≤ 0.1 nm). LPA_1_/VPC32183 (S) complex on the other hand showed classical inactivation pattern as structures initially in the intermediate state of activation were driven back to full inactivation with reformed ionic lock (TM3-TM6 > 1.0 nm) while maintaining the inactive NPxxY motif. In contrast, LPA (18:1) and LPA (20:4) preferentially drove LPA_1_ to sample active conformation with fully broken TM3-TM6 ionic lock and deviation of NPxxY motif from the inactive state (Fig. 1b). Furthermore, network analysis provided further evidence in support of TM3-TM6 ionic lock disintegration in the presence of LPA-type agonist but not VPC32183 (Fig. 1c, *i–iv*) as the edges connecting I3.52 to T6.29 was present in VPC32183/LPA_1_ complex (Fig. 1c, *i*) but not in LPA20:4/LPA1 complex (Fig. 1c, *iv*) indicating that in 80% of the simulation time, the ionic lock remained broken.

Another GPCR activation feature currently gaining research attention is the formation of a continuous and highly ordered internal water pathway^25^,^26^. To establish that this feature is present during LPA_1_ activation by LPA-type agonists, internal water molecules within the receptor were analyzed using structures generated within the last 50 ns. Interestingly, activation-dependent internal water density pattern was observed; in activated LPA_1_ (bound to LPA 20:4, LPA 18:1, AGP 18:1), we observed a continuous internal water density from the extracellular region to the cytoplasmic region through the NPxxY (Fig. 1d, *i–iii*) motif but not through membrane as observed in NECA/A_2A_R^25^. The flow of internal water is largely discontinuous in weak agonist (LPA 16:0; Fig. 1d, *iv*) and VPC32183 bound states (Fig. 1d, *v*).

The order and distribution of internal water in activated GPCR is linked with rotameric changes in the tyrosine residue of NPxxY motif^25^ and that in turn is modulated by the torsional changes in the conserved proline residue (P7.50). Here, we showed that P7.50 exhibited two-state side chain dihedrals (χ1 and χ2) conformation. In apo-LPA_1_ and weak LPA-type agonist (LPA 16:0, LPA 18:0 and LPA 14:0) and VPC32183-bound LPA_1_, metastable structures generated over the 200 ns production simulation showed that P7.50 conformations were χ1/χ2 (−20^o^, −30^o^, P^a^) and χ1/χ2 (+25^o^, +20^o^, P^b^) dihedral angle combinations separated by an energy barrier (≥+10 Kj/mol). In contrast, highly potent LPA-type agonists (LPA 20:4, LPA 18:1 and AGP 18:1) preferentially allow sampling of χ1/χ2 (−20^o^, −30^o^, P^a^) which may represent a key activation feature in LPA_1_ (Fig. 1e, *i–viii*).

### Lysophosphatidic acid-type agonist interacts with N-terminal K39 during Classical LPA_1_ activation

**Figure 2:**
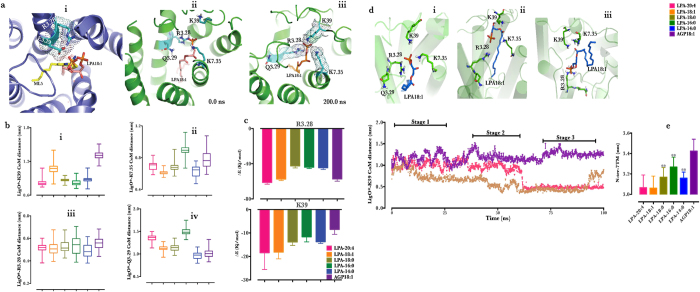
N-terminal Lys39 is involved in LPA-type agonist binding during LPA_1_. (**a**, *i*) Location of LPA18:1 (brown stick) relative to ML5 (yellow stick) in S1PR1 (purple cartoon) structure. (**a**, *ii,iii*) Location of LPA18:1 (brown stick) relative to Lys39, R3.28, Q3.29 and K7.35 (cyan stick) in the starting LPA_1_ (0 ns) and after 200 ns simulation respectively. (**b**, *i–iv)*. Box-and-Whisker plots of the center of mass distance between the head group oxygen atoms of LPA-type agonist and designated residues. (**c**, *upper and lower panels*). Contribution of R3.28 and Lys39 to LPA-type agonist binding energy respectively. (**d**) Time dependent evolution of distance between ligand head-group oxygen atoms and Lys39 during MD simulations. (inset *i–iii*) represent representative structure of LPA and LPA_1_ with emphasis on the positions of LPA18:1 relative to Lys39 (green stick) during the course of the simulations. (**e**) Center of mass distance between N-terminal residues and the 7TM bundle (see text for more description). **Data represent last 50 ns of the initial 200 ns production phase simulation and n = 3. Line graph is smoothed over 20 dataset window.

From the observations above, it is evident that LPA_1_ in complex with LPA 18:1, LPA 20:4 and AGP 18:1 did show classical GPCR activation features, thus providing the basis for studying novel N-terminal residues involved in LPA_1_ activation. Next, the contribution of each amino acid to the free energy of ligand binding was calculated focusing on the N-terminal region and the three previously identified amino acids (R3.28, Q3.29, K7.36) as references^21^. The free energy profile identified Lys39 as key N-terminal amino acids contributing to ligand binding whilst validating the contributions of the reference residues (purple arrow, supplementary Fig. 1c). Next, to prove that Lys39/ligand interaction was not present in the starting structure but formed during the simulation, we showed that head-group atoms of the starting LPA-type agonists were accurately aligned with the head-group atoms of ML5 in S1PR1-bounds state and proximal to the orthosteric residues R3.28 and E3.29 (Fig. 2a, *i*)^22^,^26^. In similar fashion, the head-group atoms were also proximal to R3.28, Q2.39 and K7.35 as predicted by Valentine *et al.*^21^ but not Lys39 in the starting LPA_1_ model (Fig. 2a, *ii*). After 200 ns simulation, a common feature in all LPA-type simulation was the presence of LPA head-group inserted between triangle-shaped electron densities of R3.28 and K7.36 and Lys39; with the phosphate group making hydrogen bond interactions with Lys39 and R3.28 (Fig. 2a, *iii*). Box-and-Whisker plots affirmed that oxygen atoms of LPA head group showed residence proximal to Lys39 (<1.2 nm) comparable with K7.36 (<1.0 nm) and R3.28 (<0.6) and to a lesser extent Q3.29 (~1.5 nm) (Fig. 2b, *i–iv*).

Furthermore, we observed that while LPA-type agonist did not show a distinct binding energy pattern with regards to acyl chain length and saturation status during interaction with R3.28 (Fig. 2c, *upper panel*), it was however noted that AGP18:1 and weaker LPA-type agonists end to exhibit a slightly reduced interaction energy with Lys39 (Fig. 2c, *upper panel*), the weakest interaction was recorded for AGP18:1. Compared with LPA18:1, AGP18:1 lacks only the acyl carbonyl oxygen, but in terms of interaction with Lys39, a rather (≈−8 Kj/mol *vs.* ≈−20 Kj/mol (LPA18:1)) very weak interaction was observed. This observation prompted the thought that acyl carbonyl oxygen may also play important role in Lys39 interaction. To validate this hypothesis, two sub-systems from acyl carbonyl oxygen-containing LPA complexes (LPA18:1/LPA_1_, LPA LPA20:4/LPA_1_) and AGP 18:1/LPA_1_ complex were subjected to another 100 ns production phase molecular dynamics simulation. The center of mass distance between oxygen atoms of LPA head-group and Lys39 showed that at approximately 50 ns, acyl carbonyl oxygen-containing LPAs moved closer to Lys39 but not AGP 18:1. To provide better understanding of the dynamics of acyl carbonyl oxygen-containing LPAs, the whole trajectory was divided into three stages based on the distance of head group to Lys39. The first stage (stage 1 ~ 25 ns) evolved structures similar to LPA head group inserting into a triangle-shaped electron densities of R3.28 and K7.36 and Lys39 (Fig. 2d, *insert i*). Between 25–65 ns (stage 2), the structures began to evolve differently with the breaking of the triangle-shaped electron densities of R3.28 and K7.36 and Lys39 by LPA, which now assumed a floating position appearing closer to N-terminal cap. Acyl-carbonyl oxygen here was positioned equidistantly from Lys39 and K7.36 while the phosphate group extended towards R3.28 (Fig. 2d, *insert i*i).

Another variant of stage 2 structures was observed in the last stage (stage 3, 70 ~ 100 ns), here, LPA completely floated away from the intra-helical cavity pushing its tail to the N-terminal cap whilst maintaining the carbonyl group proximal to Lys39 and K7.36 and its phosphate group bound to R3.28 (Fig. 2d, *insert iii*). To identify the role of carbonyl oxygen/Lys39 interaction, we speculated its involvement in N-terminal cap formation which has been reported in rhodopsin^27^. When the center of mass (CoM) distance between the N-terminal residues (1–42) and the heptahelical bundle (TM1 (Y2.41-F2.62), TM2 (Y2.41-Y2.62), TM3 (W3.25-T3.35), TM4 (V4.42-M4.56), TM5 (Y5.39-F5.62), TM6 (M6.31-L6.57) and TM7 (F7.37-Y7.55)) was calculated for LPA_1_ bound to the agonists during the last 50 ns of the trajectories, it was observed that high potency agonists (LPA18:1 and LPA20:4) caused tighter packing (~3.1 nm) compared to weaker agonists (LPA14:0, LPA16:0 and LPA18:0) and AGP18:1 appeared least packed with CoM distance > 3.4 nm (Fig. 2e).

### Mutagenesis experiment validated the critical role of Lys39 in LPA_1_ activation

**Figure 3:**
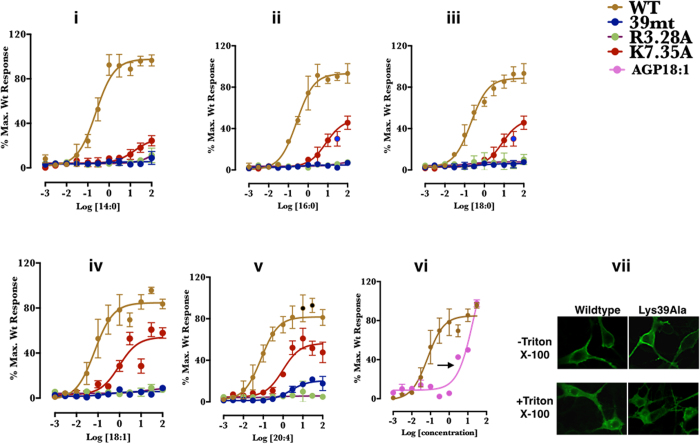
Lys39Ala LPA_1_ mutant failed to mobilize intracellular calcium in response to LPA-type agonists. (**a**, *i–vi*) dose-response curves of wildtype, Lys39Ala, R3.28A, K7.35 stimulated by various LPA-type and AGP (18:1) agonists. (*vii*). Confocal microscopy images in Triton-X100 permeabilized and non-permeabilized samples.

Till date, the most detailed study on specific residues involved in LPA-type agonist activation of the endothelial differentiation gene (EDG) family LPA receptors was reported by Valentine *et al.*^21^. By combining homology modeling, molecular docking and site-directed mutagenesis experiments, the roles of R3.28, Q3.29 were validated as LPA head-group interacting residues. Here, alanine mutant of Lys39 was constructed to validate the role of this residue in LPA_1_ under cellular condition. When B103 cells expressing normal LPA_1_, and Lys39Ala, R3.28A or K7.36A mutants of LPA_1_ were exposed to LPA14:0, intracellular calcium was poorly mobilized (<10% of the maximal response for wildtype) in all the mutants in dose-independent manner up to 100 μM (Fig. 3, *i*). When the cells were treated with LPA 16:0 and LPA 18:0, as observed for LPA 14:0, Lys39Ala and R3.28A failed to mobilize intracellular calcium up to 100 μM concentration.

LPA_1_- K7.36A-mutant expressing cells seemingly exhibited LPA-specie dependent intracellular calcium mobilization pattern. Whilst the mutation resulted in less efficient calcium mobilization across all the LPA-type agonists tested,, some LPA species tend to promote calcium mobilization at higher concentration than others. For instance, saturated LPA species (LPA 14:0, LPA 16:0 and LPA 18:0) appeared weaker (Fig. 3, *ii*,*iii*) compared to unsaturated LPA species (LPA 18:1, LPA 20:4), which exhibited dose-dependent mobilization of intracellular calcium reaching ≈60% Emax levels of the wildtype at the highest concentration tested. (Fig. 3, *iv*,*v*). If the presence of carbonyl oxygen of LPA species drives the formation of additional hydrogen bound with Lys39, its absence in alkyl-glycerol phosphate should result in lower IC_50_ value, to test this hypothesis, we compared the potencies of AGP (18:1) and LPA (18:1) in wild-type, as predicted, we observed a right-shift in the dose-response curve (Fig. 3, *vi*); suggesting the importance of the carbonyl oxygen in LPA_1_ activation. These results established that both Lys39Ala and R3.28A are critical to LPA-type agonist activation of LPA_1_. The immunoblot (supplementary Fig. 1d) demonstrates that all constructs were expressed, but LPA_1_-K39A construct expressed at a lower level than wild type and K7.36A constructs whilst the later had higher expression levels than the wild type. It is however important to note that varied receptor expression levels has negligible effect on the absolute measurement of maximal receptor activation^21^ and here, Lys39Ala had no demonstrable effect on membrane localization of LPA_1_ (Fig. 3, *vi*i) as confocal microscopy images showed a slightly increased membrane-localized LPA_1_ in comparison with the wildtype.

### LPA_1_ model well compares with ligand-binding signatures in recently resolved LPA_1_ x-ray structure

Interestingly, during the revision of this manuscript, we noticed that x-ray resolved LPA_1_- co-crystalized with three antagonists (PDB IDs: 4Z34, 4Z35 & 4Z36)^28^ were deposited in the protein data bank repository, thus allowing a more discreet discussion of our model. It is interesting to note that at all the crystal structures confirmed and ionic lock between R3.50 side chain and the carbonyl oxygen of L6.34 spaced at 5.3 Å, also, Lys39 locks the N-terminal region into close proximity with the orthosteric site for partnering R3.28 (8.5 Å)/Q3.29 (9.2 Å) during ligand binding. LPA_1_ co-crystalized with 3-{1-[(2S,3S)-3-(4-acetyl-3,5-dimethoxyphenyl)-2-(2,3-dihydro-1H-inden-2-ylmethyl)-3-hydroxypropyl]- 4-(methoxycarbonyl)-1H-pyrrol-3-yl} propanoic acid (PDB ID: 4Z35) showed a pose that drives home our point more succinctly; the propanoate moiety engages Lys39 in a salt-bridge interaction (2.8 Å), which is synonymous with the orientation of the LPA 18:1 reported here (Fig. 2a, *iii,* Fig. 2d, *i*) whilst its 4-methoxycarbonyl oxygen engages R3.38 (4.5 Å). Furthermore, the role of K7.35 in ligand binding is also confirmed in the LPA_1_ co-crystalized with 1-(4-{[(2S,3R)-2-(2,3-dihydro-1H-inden-2-yloxy)-3-(3,5-dimethoxy-4-methylphenyl)-3-hydroxypropyl] oxy} phenyl) cyclopropanecarboxylic acid, where the cyclopropanecarboxylate group formed a salt-bridge with K7.35 (2.8 Å). This structure also reiterates the importance of Lys39/R3.28 partnership as Lys39 appeared bridged to R3.28 by two crystal waters (PDB ID: 4Z36). The crystal structures also affirmed the role of internal waters in LPA_1_ (Fig. 1d, *i–v*), a non-continuous internal water signature is observable in two of the three structures (4Z35&4Z36) crystallized and one of the three crystal waters is located almost mid-way the helical bundle length in Z-direction and proximal to the NPXXY motif. A major insight that these structures provided was the role of R115 (extracellular loop 1 residue), previously, we had prepared a model where R115 was oriented towards the helical center allowing an interaction with the ligand; our site-directed mutagenesis however showed that B103 cells expressing R115A mutant showed higher potency than the wild-type (supplementary Fig. 1e, *i,ii*) with this data, we felt the ICL-1 may have been trapped by the N –terminal, thereby necessitating choosing a new starting model of LPA_1_ without the entrapment. Ultimately, the new model showed that R115 flipped outside the helical bundle into the membrane region interacting with membrane lipids, thus accounting for ~0.0 Kj/mol ligand-binding energy contribution (supplementary Fig. 1c, black arrow). Indeed, all the three structures of LPA_1_ showed outward oriented R115. Ultimately, when the 4Z34 structure was compared with our starting model, 1.736 Å alpha carbon rms value was obtained (supplementary Fig. 1f, *i,ii*).

## Discussion

The present work has identified Lys39 (N-terminal residue) as partner with R3.28, Q3.29 and K7.36 during LPA-type agonist LPA_1_ activation. In the absence of LPA_1_ crystal structure, when this experiments were performed, a starting model was built based on previous reports from our group (*accurate modeling of N- and C- termini of GPCRs using combined ab-initio and homology modeling methods*)^20^ and that of others including: Fujiwara *et al. (studied regioisomeric selectivity in LPA receptors)*^29^, Valentine *et al. (identified R3.28 and Q3.29 as key LPA*_*1*_ *orthosteric site residues)*^21^ and Hanson *et al. (2.8* Å *resolution S1PR1 receptor for modeling LPA*_*1*_ *seven transmembrane (7TM) helices)*^22^. It is worth noting that the initial model was largely inactive as its 7TM helices were modeled using S1PR1 (PDB ID 3V2Y)^22^ template with four key features: 1). The C- and N- termini were built using our recently published combined *ab initio* and homology-based modeling method^20^. 2) In compliance with cystine disulfide found in the ECL-II region of S1PR1^22^, we treated Cys^188–195^ Cys as putative cystine based on cystine-like dihedrals angles and <0.4 nm separation distance of the sulfhydryl groups during the preliminary stages of Apo-LPA_1_ MD simulation (data not shown). 3). The overall adopted ECL-II conformation for the starting model was guided by rmsd similarity with the recently published LPA_1_-ECL-II NMR structure (PDB ID: 2LQ4)^30^ and 4). Although the initial model was inactive and when compared to one of the recently published LPA_1_ structure (4Z34), our model exhibited Cα-rms value of 1.74 Å. The first MD experiment was performed on Apo-LPA_1_ in a membrane model in order to drive our model towards near native and active receptor conformation based on TM3-TM6 ionic lock rupture and TM7-NPxxY motif rmsd to inactive S1PR1 (PDB ID 3V2Y)^23^.

Our result showed that in apo-state, LPA_1_ sufficiently sampled metastable intermediately active conformations with broken TM3-TM6 ionic lock but largely inactive TM7-NPxxY motif. When LPA-type agonists were docked into the semi-active receptors such that the phosphate head-group atoms were located at hydrogen-bond-forming distance from the previously identified orthosteric residues (R3.28 and Q3.29)^21^, the receptors began to evolve structural signatures similar to activated class-A GPCRs such as increased cytoplasmic TM3-TM6 distance^23^, kinking of the 7TM^26^, and formation of a well-ordered internal water molecules^25^. These activation signatures are more robustly observed in high affinity LPA-type agonists (LPA 18:1 and LPA 20:4) compared to weak agonists (LPA 14:0, LPA 16:0 and LPA 18:0). In contrast, antagonist-bound LPA_1_, reformation of TM3-TM6 ionic lock and discontinuous internal water flow were observed.

More importantly, Lys39 was identified as indispensible to LPA_1_ activation via interaction with phosphate head-group or acyl carbonyl oxygen atom of LPA-type agonists. AGP 18:1 lacking carbonyl oxygen atom exhibited reduced residence with Lys39 and showed lower interaction propensity based on free energy calculations. Indeed, our data may provide an insight into the findings of Williams *et al.*^31^ who measured the EC_50_ values of alkylglycerol-3-phosphate (18:1, AGP) acting on LPA_1_ at 1.5 μM compared to 0.13 μM for oleoyl-LPA. Since AGP 18:1 only differs from LPA 18:1 by the absence of the acyl carbonyl oxygen atom, this data therefore supported that carbonyl oxygen forms critical interaction with LPA_1_ and Lys39 may represent one of the residues under physiological conditions.

The mutagenesis data presented also supported that Lys39 may partner with R3.28, Q3.29 and K7.36 during LPA_1_ activation as all the LPA-type agonists tested failed to mobilize intracellular Ca^2+^ in cells transiently expressing LPA_1_-Lys39Ala mutant as similarly observed in B103 cells expressing LPA_1_-R3.28Ala mutant^21^,^29^. Intracellular calcium activation in LPA_1_-K7.36Ala expressing cells was also reduced. While the roles of R3.28, Q3.29 and K7.36 as orthosteric site residues are supported by previous studies^21^,^29^, the crystal structure of S1PR1 (conserved R3.28 and E3.29)^22^ and stable residence of the side chain atoms within hydrogen bond cut-off distance from the head-group of LPA-type agonists during the simulation, what roles Lys39 may play is highly speculative, an explanation may be an interacting LPA-carbonyl-oxygen/Lys39 acting as a molecular spring, which brings the N-terminal, cap closer to the orthosteric site or GPCR bundle since further simulation experiments showed a floating LPA-type agonist towards the N-terminal cap and a well-formed LPA-carbonyl-oxygen/Lys39 with the head-group extending towards R3.28/Q3.29. Interestingly, carbonyl oxygen-containing LPA-type agonists formed a more compact N-terminal/7TM bundle compared with AGP18:1, which lacks this critical oxygen atom. N-terminal cap has been reported in a few class-A GPCRs^27^,^33^,^34^. Furthermore, it is safe to speculate that while the absence of this carbonyl oxygen may not abrogate agonist action in LPA-type agonists, it is important to further investigate its absence on LPA_1_ activation efficiency.

In conclusion, homology modeling using the structure of S1P receptor 1, *ab initio* protein-modeling and molecular dynamics simulation have provided starting model for studying the contribution of LPA_1_ N-terminal residues to LPA-type agonist interaction. Analysis of trajectories revealed that LPA-type agonists preferentially drive classical GPCR activation signatures within nanosecond simulation time period. During agonist binding and receptor activation, N-terminal Lys39 may partner with previously identified residues (K7.36, R3.28 and Q3.29). The agonists evolved two types of interaction; *phosphate head-group/Lys39* and *carbonyl-oxygen/Lys39*. When LPA-type agonist via carbonyl-oxygen, N-terminal cap closure may be facilitated while the phosphate head-group is allowed to freely interact with R3.28/Q3.29. Closed N-terminal cap in ligand-bound state may provide a mechanism for differentiating an occupied from a free LPA_1_.

Interestingly, LPA1 x-ray structure co-crystalized with three antagonists (PDB IDs: 4Z34, 4Z35 & 4Z36) during the revision of the manuscript confirmed that Lys39 locks the N-terminal region into close proximity with the orthosteric site for partnering R3.28 and it is of importance to note that were ligands are not directly, involved, Lys39 is bridged to R3.28 by crystal waters. The crystal structures also affirmed the role of a non-continuous internal water signature in antagonist-bound state. R115 attached to flexible extracellular loop-1 better associate with the membrane accounting for increase potency of R115A. Lys39 may be unique for LPA_1_ but other endothelial differentiation gene-class receptors have similar residues we have noticed lys34 in S1P1 crystal structure, and of particular interest is the loss of LPA_2_ activation in lys31Ala (unpublished data). Thus establishing that more research is required to fully identify the how to develop LPA_1_-specific antagonists.

## Methods

### LPA_1_ and Ligand coordinates

The structure of LPA_1_ was built as previously described with few modifications. Briefly, using the Modeller suite (*ver. 9.11*)^35^, the 7Tm helices of LPA_1_ were built from the x-ray structure of S1PR (PDB ID: 3V2Y)^22^. Based on the DOPE-HR scoring, the best three models were selected for loop refinement. The choice of a model following loop refinement was guided by the root mean square distance (RMSD) values of generated structures from the LPA_1_-ECL-II (PDB ID: 2LQ4) structure^30^. Lastly, the N- and C-termini termini were refolded using the LPA_1_ structure generated from I-TASSER/*ab initio* method as previously described^20^. The representative model was then subjected to fragment-guided molecular dynamics (FG-MD) simulation for atomic-level protein structure refinement^36^. Rotameric errors and steric clashes in the FG-MD output were checked using the protein preparation utility in Molecular Operating Environment (MOE)^37^ software followed by the protonation of titratable residues at pH 7.2 using GBSA solvation model to give the final LPA_1_ model. The model quality was checked using critical assessment of methods of protein structure prediction (CASP) protocol^38^ as implemented on the Swiss model server (http://swissmodel.expasy.org). The 2D atomic coordinates of 1-myristoyl-2-hydroxy-sn-glycero-3-phosphate (LPA14:0) (857120), 1-palmitoyl-2-hydroxy-sn-glycero-3-phosphate (LPA16:0) (857123), 1-stearoyl-2-hydroxy-sn-glycero-3-phosphate (LPA 18:0) (857128), 1-oleoyl-2-hydroxy-sn-glycero-3-phosphate (LPA18:1) (857130), 1-arachidonoyl-2-hydroxy-sn-glycero-3-phosphate (ammonium salt)(LPA20:4), (S)-phosphoric acid mono-(2-octadec-9-enoylamino-3-[4-(pyridin-2-ylmethoxy)-phenyl]-propyl) ester (VPC 32183 (S)) (857340), were obtained from the AVANTI lipids website (http://avantilipids.com) and alkyl glycerol phosphate 18:1 (AGP 18:1) coordinate was generated from LPA18:1 via removal of the carbonyl oxygen using ChemAxon software (http://www.chemaxon.com).

### Biosystems setup

To generate LPA_1_ in complex with a ligand, first, S1PR (PDB ID: 3V2Y) 3D structure was superimposed on LPA_1_ structure thus, allowing the visualization of ML5 (ligand) within LPA_1_. With the deletion of S1PR1 coordinate, 2D structures of the ligands were virtually docked into the LPA_1_ using MOE dock^37^ as previously reported^39^. Orientation (along the membrane normal) of all the biosystems was performed using the PPM server (opm.phar.umich.edu/server.php)^32^. Each of the oriented biosystem was inserted into a pre-equilibrated 1,2-Dilauroyl-sn-glycero-3-phosphocholine (DLPC, 68 lipids per leaflet) bilayer using CHARMM-GUI webserver (www.charmm-gui.org)^40^. Ligand parametization was performed using ParamChem service (https://cgenff.paramchem.org) as implemented on CHARMM-GUI webserver. The biosystems were solvated in TIP3P explicit water model^41^ and neutralized with Na^+^/CL^−^ (0.15 M).

### Molecular dynamics (MD) simulation

All molecular dynamics simulation was run on GROMACS (*ver*. 4.6)^42^ software using CHARMM36 force field^43^. During equilibration, the biosystems were subjected to constant pressure and temperature (NPT; 310K, 1 bar) conditions using Berendsen temperature^44^ and pressure coupling algorithms as implemented in GROMACS. Van der Waals interactions were estimated at 10 Å, long-range electrostatic interactions were computed using particle mesh Ewald (PME) summation scheme^45^ while equation of atomic motion was integrated using the leap-frog algorithm^46^ at 2 fs time step for a total time of 100 ns with positional restraints imposed on the heavy atoms in all directions. To generate active-state apo-LPA_1_, a 150 ns unrestrained molecular dynamics simulation was performed under similar conditions above without positional restraints. Apo-LPA_1_ was adjudged active with the rupture of TM3-TM6 ionic lock and TM7-NPxxY rmsd far >0.05 nm from the inactive (see results for details). Three (n = 3) starting coordinates representing intermediately active LPA_1_ were harvested from the 3D graph apo- LPA_1_ graph (Fig. 1a) using MATHEMATICA^47^ get coordinate module. All the ligands were docked into apo-LPA_1_ as described above. Cys^188^ and Cys^195^ located on the extracellular loop II were treated as a cystine disulfide. Our choice of this combination was informed by previous reports that a distance 0.4 nm between the sulfur atoms and 90° dihedral angle are required for disulfide bond formation. Analysis of the LPA_1_/POPC production phase data showed that Cys^188^ and Cys^195^ but not Cys^188^ and Cys^190^ nor Cys^190^ and Cys^195^ fulfilled this requirements (data not shown)^48^. The complexes were equilibrated with full atomic restraints (50 ns) followed by 200 ns production simulations. A sub-set of the production simulation was repeated for some agonists (LPA 18;1, LPA20:4 and AGP 18:1) for an additional 100 ns to further study specific interactions between the ligands and LPA_1_. In all the simulations, lipid bilayer thickness was fairly maintained between 3 ~ 4 nm throughout the simulation (Supplementary Fig. 1a) while the area per lipid was maintained fairly at 80 Å^49^ (Supplementary Fig. 1b) as shown for the first 200 ns production phase of the complexes. All calculations were performed on SuperMicro workstations (32-E2600 Intel Xeon CPUs, 4 Tesla K40 GPUs Accelerator PCI-E x16 Card/node).

### Data analysis

All 3D surface graphs were plotted using MATHEMATICAL^47^ statistical software from MD simulation data calculated using in-house *ad-hoc* script and *g_sham* available in GROMACS software. Root mean square deviation (rmsd) was calculated using *g_rms* tools, *g_dist* tool was used for calculating the center of mass distance of two groups of atoms while g_angle was used to calculate χ1 and χ2 dihedral angles. Water density was calculated using Volmap plugin in visual molecular dynamics (VMD)^50^ software as previously described^25^. Binding free energy was calculated using *g_mmpbsa* algorithm as described^51^. Dynamical networks (set of nodes with connecting edges) for LPA_1_ TM helices were calculated as described^52^ using Carma (*ver. 1.4*)^53^, gncommunities and subopt scripts^52^ and implemented in VMD. Here, a pair of nodes was connected by an edge if the corresponding monomers were resident within 4.5 Å distance for at least 80% of the frames analyzed. The size of an edge corresponds to their weights. Line graphs were plotted as mean ± standard error of mean (S.E.M) from 2 ~ 3 independent runs using GraphPad prism (*ver 6.0e, 2014*). Unless otherwise stated, LPA_1_ residues are represented using Ballesteros-Weinstein nomenclature^54^.

### Site-directed mutagenesis experiments

PCXN2.1-LPA_1_, an expression vector for human LPA_1_, was kindly provided by Dr. T. Shimizu (NCGM, Tokyo) (40). The mutants of LPA_1_ (Lys39Ala, Arg115Ala, Arg124Ala, Asp191Ala and Lys294Ala) were generated by a two-step PCR. The cDNA of the 5′ portion of LPA_1_ was amplified by PCR using a forward primer containing XhoI site and reverse primers containing the sequence encoding the mutated amino acids. The 3′ portion was also amplified with forward primers containing the mutated sequence and a reverse primer containing NotI and BglII sites. All cDNAs of the LPA_1_ variants were amplified using a mixture of the two PCR products together with the XhoI and NotI-containing primers. The resulting 1.0 KB products were cloned into a pCRII-Topo vector for sequence analysis. The cDNA of LPA_1_ mutants were cut out from pCRII-Topo by XhoI and NotI, and ligated into the XhoI/NotI sites of pCAGGS-HA (41), to generate pCAGGS-HA-LPA_1_ mutants.

### Cell culture and intracellular calcium ion (Ca^2+^) mobilization assay

B103 rat neuroblastoma cells that lack LPA response were cultured in DMEM containing 10% fetal bovine serum at 37 °C in a 5% CO_2_ atmosphere. Transfection method for LPA_1_ (and mutants) into B103 cells, and intracellular calcium ion mobilization assay protocol have been previously described^55^. Briefly, B103 cells (1 × 10^7^) were transfected with plasmid encoding either wild type or each mutant receptor using NEPA21 Super Electroporator (Nepa Gene, Tokyo, Japan). After 24 h of transfection, the cells were harvested by centrifugation and suspended with 0.1% BSA supplied-DMEM. The cell suspension was plated in a 384-well plate with the density of 5 × 10^3^ cells/well. Following incubation for 18–24 h, cells were loaded with 10 μl Fluo-8 (8 μM) in 0.1% BSA supplied-DMEM containing 1 mg/ml amaranth. After 30 min, 20 μl of the LPA species at defined concentration was added followed by an immediate recording of the fluorescence using the Functional Drug Screening System/μCell (Hamamatsu Photonics K.K., Hamamatsu city, Japan). The fluorescence intensity was described as fura-2 ratio (tested value/basal value) or fold induction. Dose-response curves were plotted as mean ± S.E.M of at least two (2) independent experiments using GraphPad prism^56^.

### Immunoblot of wildtype LPA_1_ and mutant receptor types

B103 cells transfected with either wildtype or mutant LPA_1_-containing plasmid were seeded at 1 × 10^4^ cells/cm^2^ onto an 8-well slide glass coverslips coated with collagen (BD Bioscience, San Lose, CA, USA). The cells were cultured for another 24 h. For immunoblot analysis, total protein (8 μg) of B103 cells transfected with HA-tagged wildtype or mutant LPA_1_ was separated on SDS-polyacrylamide gels (10%). Primary antibodies were used as follows: anti-HA antibody (1:1000;Covance, CA), anti-GAPDH antibody (1:1000; Millipore) and anti-Actin antibody(1:1000; Millipore). Horseradish peroxidase-labeled anti-mouse IgG was used as a secondary antibody at a dilution of 1:1000. Immunoreactive bands were detected using an enhanced Chemiluminescent Substrate (SuperSignal West dura Chemiluminescent substrate, Pierce Chemical, Rockford, IL) for the detection of horseradish peroxidase.

#### Confocal microscopy of wildtype and Lys39Ala LPA_1_

B103 cells transfected with either wild type or mutant LPA_1_-containing plasmid were seeded at 5 × 10^4^ cells/well onto an 8-well slide glass coverslips coated with lysine. The cells were cultured for another 24 h followed by incubation in 4% paraformaldehyde for 30 min at 25 °C. Fixed cells were rinsed three times with TBS for non-permeabilization or with Triton X-100 (0.1% in TBS for 3 minutes) for permeabilization. Immunofluorescence labeling was performed by blocking the sections with 1% BSA in TBS or TBST for 1 h at room temperature followed by incubation with anti-HA antibody (1:300; Covance, CA) overnight at 4 °C. After washing with TBS or TBST, sections were incubated with Alexa Fluor 488-conjugated anti-mouse IgG (1:300; Invitrogen, Carlsbad, CA) for 2 h at room temperature. After washing, the sections were cover-slipped with VECTASHIELD mounting medium (Vector Laboratories, Inc., Burlingame, CA) and examined using an LSM 710 confocal microscope with ZEN Software (Carl Zeiss, Oberkochen, Germany).

## Additional Information

**How to cite this article**: Omotuyi, O. I. *et al.* Lys39-Lysophosphatidate Carbonyl Oxygen Interaction Locks LPA_1_ N-terminal Cap to the Orthosteric Site and partners Arg124 During Receptor Activation. *Sci. Rep.* **5**, 13343; doi: 10.1038/srep13343 (2015).

## Supplementary Material

Supplementary Information
