# Supplementary material for: Lys39-Lysophosphatidate Carbonyl Oxygen Interaction Locks LPA1 N-terminal Cap to the Orthosteric Site and partners Arg124 During Receptor Activation
Source: Sci Rep. 2015 Aug 13;5:13343. doi: 10.1038/srep13343 (PMC4542628; doi:10.1038/srep13343)
Supplement: Supplementary Information [file srep13343-s1.pdf]

Supplementary information

Lys39-Lysophosphatidate Carbonyl Oxygen Interaction Locks LPA_1 N-terminal Cap to the Orthosteric Site and partners Arg124 During Receptor Activation

Olaposi I. Omotuyi[1,2] Jun Nagai[1], and Hiroshi Ueda[1,2]

[1]From the Department of Pharmacology and Therapeutic Innovation,

Graduate School of Biomedical Sciences, Nagasaki University, Japan.

[2] From the Center for Drug Discovery and Therapeutic Innovation, Nagasaki University, Japan.

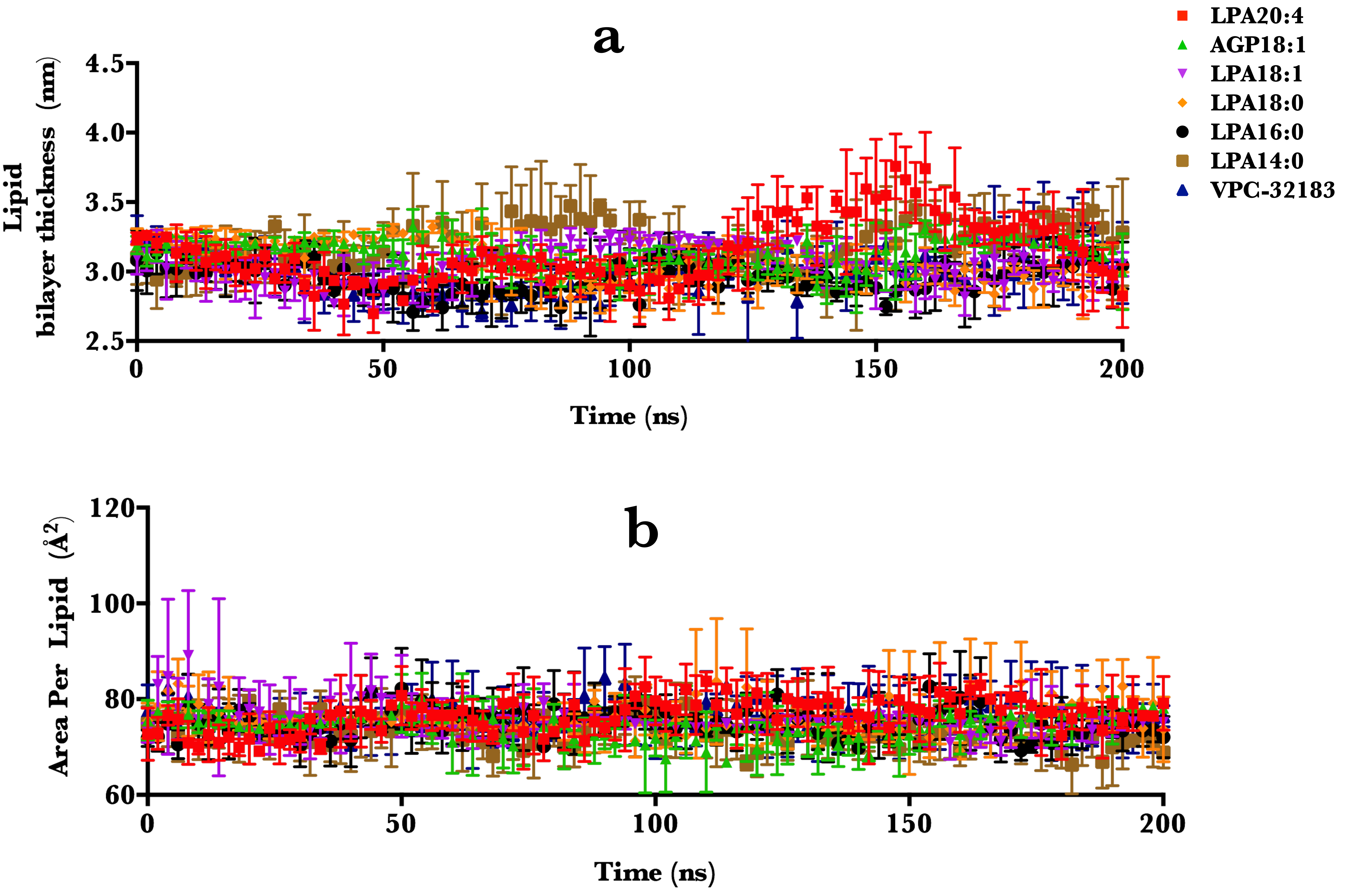

a
Lipid bilayer thickness (nm)
4.5
4.0
3.5
3.0
2.5
0
50
100
150
200
Time (ns)
LPA20:4
AGP18:1
LPA18:1
LPA18:0
LPA16:0
LPA14:0
VPC-32183
b
Area Per Lipid (Å2)
120
100
80
60
0
50
100
150
200
Time (ns)

c

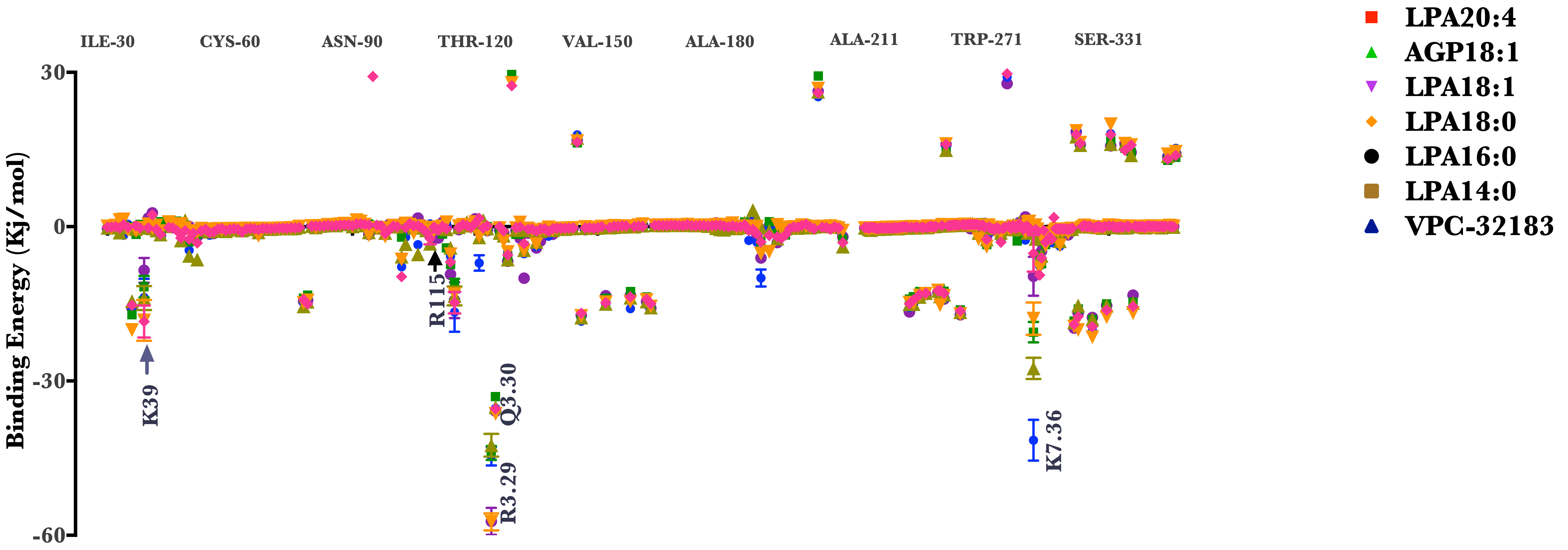
ILE-30
CYS-60
ASN-90
THR-120
VAL-150
ALA-180
ALA-211
TRP-271
SER-331
Binding Energy (Kj/mol)
30
0
-30
-60
K39
R115
Q3.30
R3.29
K7.36
LPA20:4
AGP18:1
LPA18:1
LPA18:0
LPA16:0
LPA14:0
VPC-32183

d

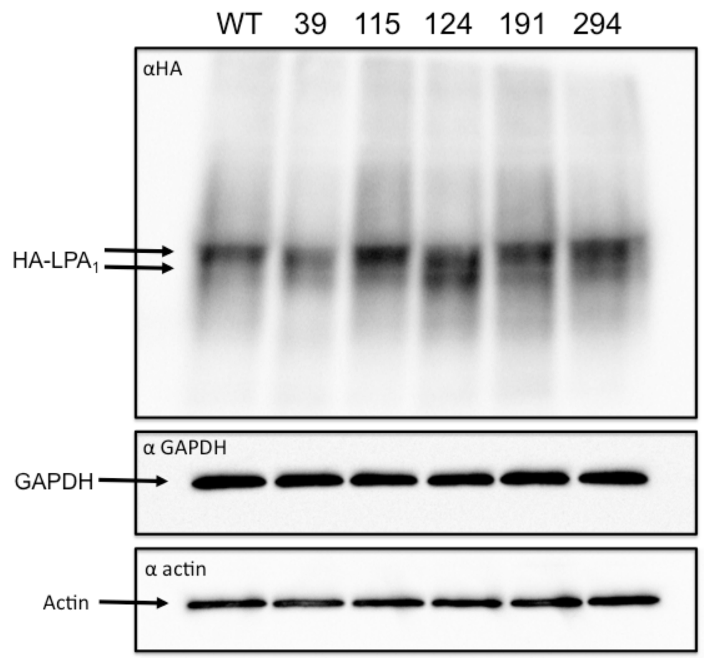
WT
39
115
124
191
294
αHA
HA-LPA1
α GAPDH
GAPDH
α actin
Actin

e

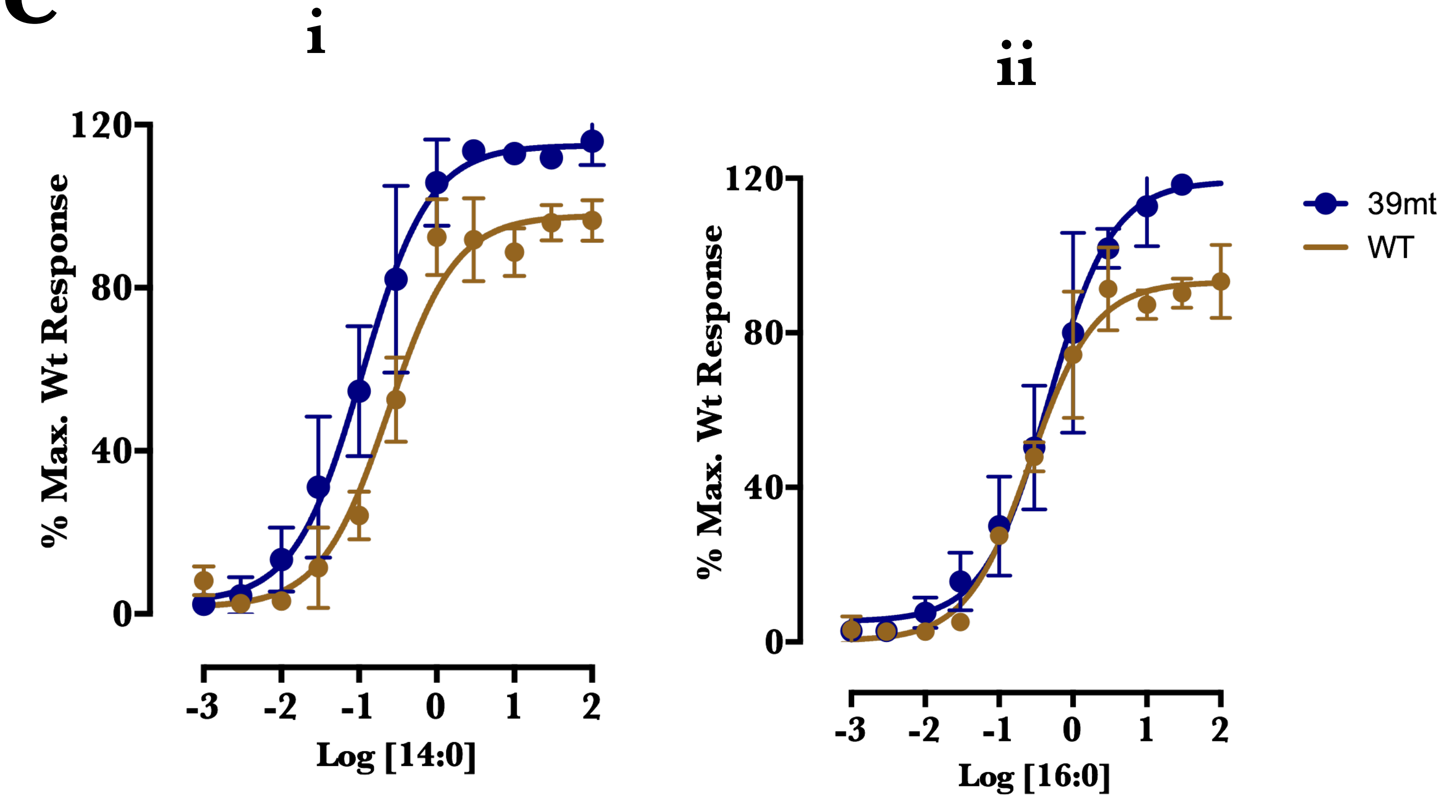
i
ii
% Max. Wt Response
120
80
40
0
-3
-2
-1
0
1
2
Log [14:0]
Log [16:0]
39mt
WT

Supplementary fig. 1. (a,b) Time dependence of lipid bilayer thickness and area-per-lipid during MD simulations. (c) Energy profile of LPA_1 amino acids (last 50 ns of the simulation). (d) immunoblot signals of wildtype, Lys39Ala, R3.28A, K7.35 and randomly selected Arg115Ala and Asp191Ala. (Randomly selected mutants served as controls, all blots are from the same run, the internal controls (higher constrats were applied to internal control (GAPDH and Actin) bands for imporved visibilities. LPA_1 bands were cropped on the thick black rectangular border to emphasis a single band without background constrast). (e, *i-ii*), Dose-response curves of wildtype and R115Ala LPA_1 in response to LPA species. (f, i-*ii*) Superimposed staring LPA_1 structure and recently crystallized 4Z34 (red and green cartoons represent 4Z34 and LPA_1 model respectively).
